# PKreport: report generation for checking population pharmacokinetic model assumptions

**DOI:** 10.1186/1472-6947-11-31

**Published:** 2011-05-16

**Authors:** Xiaoyong Sun, Jun Li

**Affiliations:** 1 Bioinformatics and Computation Biology Program, Department of Statistics, Iowa State University, Ames, Iowa 50011, USA; 2 Facult é de Pharmacie, Université de Montréal, C.P. 6128, Succ. Centre-ville, Montréal (Québec), H3C 3J7 Canada; 3 Centre de Recherche Mathématiques, Université de Montréal, C.P. 6128, Succ. Centre-ville, Montréal (Québec), H3C 3J7 Canada

## Abstract

**Background:**

Graphics play an important and unique role in population pharmacokinetic (PopPK) model building by exploring hidden structure among data before modeling, evaluating model fit, and validating results after modeling.

**Results:**

The work described in this paper is about a new R package called PKreport, which is able to generate a collection of plots and statistics for testing model assumptions, visualizing data and diagnosing models. The metric system is utilized as the currency for communicating between data sets and the package to generate special-purpose plots. It provides ways to match output from diverse software such as NONMEM, Monolix, R nlme package, etc. The package is implemented with S4 class hierarchy, and offers an efficient way to access the output from NONMEM 7. The final reports take advantage of the web browser as user interface to manage and visualize plots.

**Conclusions:**

PKreport provides 1) a flexible and efficient R class to store and retrieve NONMEM 7 output, 2) automate plots for users to visualize data and models, 3) automatically generated R scripts that are used to create the plots; 4) an archive-oriented management tool for users to store, retrieve and modify figures, 5) high-quality graphs based on the R packages, lattice and ggplot2. The general architecture, running environment and statistical methods can be readily extended with R class hierarchy. PKreport is free to download at http://cran.r-project.org/web/packages/PKreport/index.html.

## Background

The application of population pharmacokinetic (PopPK) modeling in the drug development has grown in this decade. It has numerous advantages over non-compartmental analysis: incorporating unbalanced designs, modeling sparse data [1-3] and quantifying individual variability. However, these advantages increase the complexity of model bringing additional consideration to the results, and more difficulties in checking how well the model fits the data. This paper describes an R package for generating reports for PopPK models, that contain comprehensive summary statistics and graphics. Graphics play an important and unique role in PopPK model building through exploring hidden structure among data before modeling, evaluating model fit, and validating results after modeling [4-13].

The output of PKreport follows many of the recommendations in Ette's comprehensive tutorial on the application of graphics in PopPK modeling [8]. By exploring distribution plots, scatter plots, residual plots, partial residual plots, pairs plots, conditional plot, contour plots and start plots, he extensively demonstrated the graphic ability in the field of PopPK. At the same time, from a model perspective Karlsson investigated assumption testing comprehensively for PopPK model based on graphics [7]. In that paper, the authors described 22 assumptions for various situations during the model development. By going through each stage of model building process with graphics, Bonate gave a detailed demonstration on how to facilitate model building with graphics, especially with real PopPK examples [14].

In 1999, as a continuation of the work in 1998, Jonsson developed a software tool: Xpose to help model building with graphics [15]. Equipped with data set checkout plots, goodness of fit plots and tools for covariate model selection, this software has gained great popularity. Later, Wilkins further created a graphical user interface and management tool: Census, to help Xpose diagnose models [16]. In 2003, Monolix was developed as a Matlab program. Compared with NONMEM, it employed an alternative approach to calculate maximum likelihood estimators based on SAEM algorithms [17]. Monolix provides user-friendly graphical interface, powerful and convenient PK/PD model library, goodness of fit plots, and a stand-alone non-matlab program. PKreport further advances this work by providing automatically generated routine graphics, as required for example by the Federal Drug Administration (FDA).

PKreport provides 1) a flexible and efficient R class to store and retrieve NONMEM 7 output, 2) automate plots for users to visualize data and models, 3) automatically generated R scripts that are used to create the plots, that can be used later for reproducing the same or specific results, 4) an archive-oriented management tool for users to store, retrieve and modify figures, 5) high-quality graphs based on the R packages, *lattice *[18] and *ggplot2 *[19]. The general architecture, running environment and statistical methods can be readily extended by the user.

The paper is organized as follows. The following section explains the methods implemented in the report. The third section focuses on the software implementation. The fourth section demonstrates how to use this package. The fourth section discusses the unique features of this package. The conclusions and future work are discussed in the final section.

## Methods

Many authors [7,8] have done extensively research in model assumption testing, and we follow these guidelines to automatically perform the following assumption testing: 1) exploratory data analysis; 2) goodness of fit plots; 3) parameter and random effects evaluation; 4) structural model diagnostics; 5) residual model diagnostics; 6) covariate model diagnostics. PKreport can be run on these subsets of methods, or on everything.

### Exploratory data analysis

Dose history, covariate information, and diverse clinical trials taken in different arms or different periods should be checked for correctness and accuracy before models construction. Data structure should be investigated to screen hidden patterns, outliers and extreme observations linked to individuals for further analysis. Currently, histogram and scatter plot combined with conditional plot are implemented to help achieve these goals. Karlsson emphasized the plots for each patient ID versus each variable in the data file [7], and Ette described exploratory examination of concentration, distribution and correlations between covariates [8]. All of these guidelines have been implemented in the PKreport package.

### Goodness of fit plot

Goodness of fit plot plays a key role in checking model fitting. These kinds of plots give an overall perspective of model performance, including scatter plots for concentration versus PRED, concentration versus IPRED, PRED versus IDV and IPRED versus IDV [20]. Most reports submitted to FDA are required to explain response from each patient. Individual plots for concentration/PRED/IPRED versus IDV can be explored for this purpose.

### Evaluate parameters and random effects

Generally, there are assumptions for distribution of parameters during modeling process. The histogram is utilized to check this distribution. In addition, the correlation of parameters (clearance, volume distribution, etc) has significant effect on modeling performance, and it is checked by scatter plots or a scatterplot matrix. The assumptions for random effects are also tested for distribution and correlation by histogram, scatter plots or a scatterplot matrix.

### Diagnose structural models

Structural model describes the model without the covariates. In practice, there are three popular structural models for use, including 1-, 2-, and 3-compartment models with different absorption models. After determining structural models, we can further build covariate models by incorporating relevant covariates. Structural model is diagnosed by PRED versus concentration conditioned on time, IPRED versus concentration conditioned on time, WRES versus time, WRES versus PRED, PRED versus concentration conditioned on covariates, IPRED versus concentration conditioned on covariates.

### Diagnose residual error models

Residual model deals with random and unexplained variability (ε in the following function) due to model misspecification, assay errors, dosing history errors, etc.(1)

Generally, PopPK model consists of the following common residual models [5,14]:

• additive error model(2)

• proportional error model(3)

• exponential error model(4)

• combined additive and proportional error model(5)

Two assumptions are related to this submodel: 1) homoscedastic variability; 2) symmetrically distributed residuals. To test these assumptions, we apply the following techniques: 1) histogram for distributions of WRES; 2) histogram for individual distribution of WRES; 3) scatterplot of |WRES| versus PRED to check the shape of residual; 4) scatterplot of |WRES| versus PRED conditioned on covariates to screen the covariate effects; 5) autocorrelation of WRES.

### Diagnose covariate models

In general, covariate models study how to incorporate covariates into the model such that the associated variability can be reduced and the model explanation power enhanced. By linking subject-specific characteristics with model parameters, we can identify relevant covariates for model. Parameters, ETA and WRES are of great use to help screen proper covariates. We utilize the following methods to check covariate models: 1) scatter plot for parameters versus covariates, ETAs versus covariates, WRES versus covariates; 2) scatterplot matrix of covariates.

## Implementation

PKreport is an R package aiming to create an automatic pipeline for model assumption testing. Based on a hidden metric system matching default modeling variables to data variables, this package turns the assumption testing discussed in the previous sections to a fast, convenient and comprehensive routine. With the support of two powerful R graphical packages (*lattice *and *ggplot2 *), this software can generate high-quality figures for diagnosis, archive all figures with specific folders for report and review, and utilize web browser as the interface for viewing, archiving and analyzing.

### Metric system

The default modeling variables function as the currency for communicating between data sets and the package to generate special-purpose plots (Table 1). For example, PRED represents prediction calculated from nonlinear mixed effects model fitting, and RES is equal to the difference between observations and predictions [11,21]. Users may use preferred modeling software to calculate these related variables. As a result, each data set and fitting results have totally different variable names for further analysis. To facilitate model diagnostics, users need to match the package metric system with the variables from output obtained from the modeling software. After matching, the package can process data, configure functions, and generate related diagnostic plots. This system provides ways to function for diverse software such as NONMEM, Monolix, R, and SAS.

**Table 1:** Package metric system

Package variable	Description
ID	Patient ID
TIME	Time after dose
CONC	The concentration of drug in the body
PRED	Prediction generated from model fitting
RES	Residual
WRES	Weighted residual
IPRED	Individual prediction
IWRES	Individual weighted residual
COV	Covariates
PARA	Parameters, such as clearance, volume of distribution, etc.
IDV	Independent variable (usually time)

The first column (Package variable) contains default modeling variable names. These names match NONMEM naming system. The second column explains the details of these variables.

### Configuration

The whole system is configured by three lists: 1) graph list. This list helps the user to choose proper figure format (jpg, pdf, png, etc.) as well as the graphical packages. Currently there are two popular graphical packages implemented for high-quality figures (*lattice *and *ggplot2 *). 2) histogram list. This list specifies the configuration for the histogram generated by this package. It includes type of histogram and layout setup. 3) scatterplot list. This list determines type of scatter plot, bandwidth of smooth and layout setup.

### Architecture description and features

**Figure 1:**
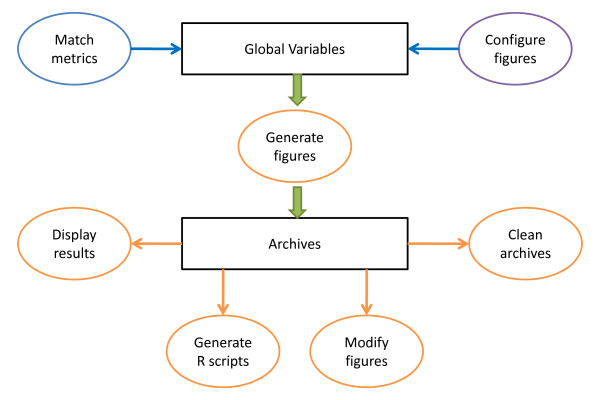
**Software architecture of PKreport**. It has seven main functions: 1) match metrics; 2) configure figures; 3) generate figures; 4) display results; 5) generate R scripts; 6) modify figure; 7) clean archives. The first two functions set up working environment, and the other functions help to generate reports.

Currently PKreport only offers console user interface to test model assumptions. It has the following functions: 1) Match metrics. By matching default package variables to data variables based on one-to-one or one-to-many schema, this function sets up global variables for further analysis. 2) Configure figures. This module determines the figure format, figure size and other related properties of figures. 3) Generate figures. Depending on the research goals, users have access to 7 sub-functions for exploratory data analysis, overall goodness of fit plots, parameter diagnostics, random effects diagnostics, structural model diagnostics, residual model diagnostics, and covariate model diagnostics. Each sub-function will create a folder to store related figures as archives. 4) Display results. PKreport offers web browser as a management tool to explore the archives created in function 3 and R scripts in function 5. The main interface includes the names of file directories. 5) Generate R scripts. To improve efficiency and help users to generate high-quality figures, users have option to modify related R scripts to meet their specific requirements. All generated R scripts match the order of figures generated in function 4. 6) Modify figures. Users can also update or modify certain figures with figure ID for particular purpose. 7) Clean archives. This module will delete all archives (file directories and figures) and clean the global variables in R environment. The general architecture is shown in Figure 1.

### nonmem class

R supports two different object-oriented programming approaches: S3 and S4. *nonmem *is an R class, and it implements a S4 class hierarchy. The goal of this class is to provide an efficient way to access the output from NONMEM 7 in R. It stores lst, tab, cov, cor, coi, and phi files in R environment with the flexible structure based on object-oriented programming (OOP). This class includes the following slots: file.lst, method, analysis, objt, objv, objs, tabid, tabdata, file.cov, file.cor, file.coi and file.phi (Table 2). Additionally, this class has the following methods: non.list, non.list.meth, non.lst.term, non.lst.objt, non.lst.objv, non.lst.objs, non.tab, non.cov, non.cor, non.coi, non.phi. In this version, nonmem class does not support multiple estimation method of NONMEM 7 yet.

**Table 2:** Design of nonmem class in R

*nonmem *class slots	*nonmem *class methods	Storage mode	Description
file.lst	non.lst, non.select	character	Standard output in **lst file**.
method	non.lst.meth	character	Estimation method extracted from #METH tag in **lst file**.
analysis	non.lst.term	list	Analysis information extracted between #TERM and #TERE tag in **lst file**.
objt	non.lst.objt	character	Objective function extracted from #OBJT tag in **lst file**.
objv	non.lst.objv	character	Objective function value extracted from #OBJV tag in **lst file**.
objs	non.lst.objs	character	Objective function standard deviation extracted from #OBJS tag in **lst file**.
tabid	non.tab	character	The title of tab file (the first line of the file) in **tab file**.
tabdata	non.tab	data frame	Output data starting from the second line in **tab file**. This is the main data for PKfigure.
file.cov	non.cov	list	Title (character) and data (data.frame) in **cov file**.
file.cor	non.cor	list	Title (character) and data (data.frame) in **cor file**.
file.coi	non.coi	list	Title (character) and data (data.frame) in **coi file**.
file.phi	non.phi	list	Title (character) and data (data.frame) in **phi file**.

The first column describes the slots in *nonmem *class, the second column explains the related methods to access the slots, the third column is the storage mode for the slots, and the last column gives the detail of the slot contents.

### Figure archives

The package will automatically store figures generated from graphic reports in the file system. The figures are categorized by the model diagnostics methods. If all methods are utilized for report, nine folders will be created with the proper figures. "univar" and "bivar" folders are for exploratory data analysis; "gof" folder is for goodness of fit; "struct" folder is for structural model diagnostics; "resid" folder is for residual model diagnostics; "para" folder is for parameter diagnostics; "cov" folder is for covariate model diagnostics; "eta" folder is for random effects diagnostics and "ind" folder is for individual plots.

The format of figures is specified in save.format option in PKconfig function, and currently it supports png, bmp, jpeg, and tiff. png files are automatically generated for html report. After analysis, the figures will be stored in the proper folders with the specified file formats.

The figure archives can be deleted with PKclean function. During analysis, if users work through the diagnostic method step by step, the archives will be cleaned automatically unless clean option in PKfigure function is set as FALSE.

### Report format

**Figure 2:**
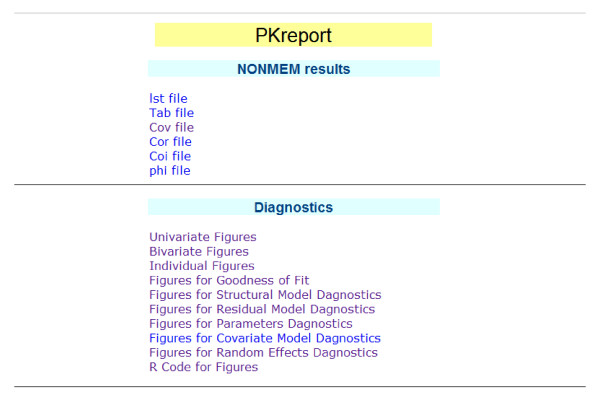
**PKreport web interface**. The first section: NONMEM 7 result is numerical report. The second section: Diagnostics is the graphical report. The R code is the last section of graphical report.

**Figure 3:**
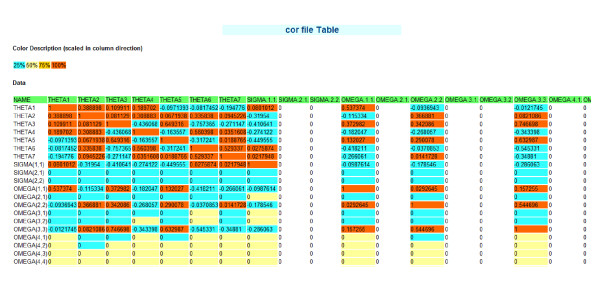
**Numeric report for cor file**. The values in each column/variable are colored by lower quartile, median and upper quartile. The values below 25% are labeled with light blue, the values between 25% and 50% are labeled with yellow, the values between 50% and 75% are labeled with orange and the values above 75% are labeled with red. The columns/variables with constant numbers are left without coloring. Note: the color schema for the values is annotated on the top of the table.

The reporting system in this package includes two types of report: numeric report and graphical report (Figure 2). Both reports are html reports and employ web browser as the user interface. Numeric report is only designed for NONMEM 7 (Figure 3). It generates the heatmap-like tables for tab, cor, cov, coi and phi files. The values in each column (variable) are colored by lower quartile, median and upper quartile. The columns (variables) with constant numbers are left without coloring. In addition, the color schema for the values is annotated on the top of the table. Table can be bi-clustered in both row and column direction as heatmap with two arguments (table.Rowv and table.Colv) in the PKshow function. This heatmap-like table has all the advantages of heatmap. It gives users an eye view of similar patient groups (clustered by row), similar variable groups (clustered by column) or similar bi-clustered groups (clustered by both row and column).

**Figure 4:**
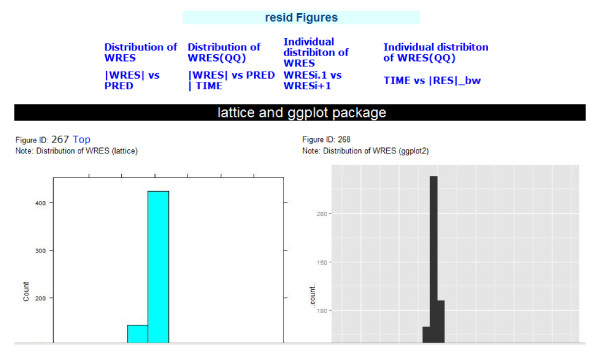
**Figures for residual model diagnostics**. A summary list for all figures is on the top. The figure ID matches the code in the R code web page, and users can easily regenerate the figure with this ID. Left figure: histogram for distribution of WRES generated with *lattice *package. Right figure: histogram for distribution of WRES generated with *ggplot2 *package.

Graphical report mainly targets model diagnostics with a series of plots for different assumption testing. Each diagnostic method has a separate html report, including all related figures (Figure 4). This html report has a summary list for all figures on the top, and every figure name in the figure achieves will match either one or two graphical packages (lattice and ggplot2). The figure ID matches the R script ID in R code web page, and users can easily regenerate the figure with this ID.

All R codes for figures are automatically generated in the report. Each R code command includes two comments and one script. The first comment explains the folder name for this figure and figure ID matching the graphical report. The second comment describes the title of the figure. The R script can be run to regenerate figures for further usage. In addition, all the R codes are stored as a text file (PKcode.txt) in the current R working directory.

### Working pipeline

This package supports a flexible pipeline for reporting and analyzing outputs from NONMEM 7. It includes data input, data configuration, model diagnostics, report generation and data cleaning.

#### Data input

The raw data can be output from NONMEM, Monolix or SAS. For NONMEM 7, this package requires standard input (lst file) and fitting results (tab file). It also works with some new files generated only for this NONMEM version, such as cor, cov, coi and phi files. For Monolix, SAS, and other version of NONMEM, this package requires only fitting results. The main function is as follows,

> myNonmemObj <- new("nonmem'',

   output.lst= "C:/nonmem7/test.lst'',

   output.tab= "C:/nonmem7/test.FIT'',

   output.dir= "C:/nonmem7'')

#### Data configuration

The objectives of this step are twofold. First, users can setup global parameters for this package. It includes graphic package choice, figure configuration and saving format. Second, users are required to link package metric system to the variables in the data for further model diagnostics.

# First: setup global configuration

> PKconfig(general.list, hist.list, scatter.list)

#Second: match metric system

> PKdata(data=pdata, match.term=var.name)

#### Model diagnostics

The main goal of this step is to generate figures for model diagnostics. It performs the following model assumption testing: exploratory data analysis, goodness of fit plots, parameter and random effects evaluation, structural model diagnostics, residual model diagnostics, and covariate model diagnostics.

# residual model diagnostics

> PKfigure(pdata, 5)

#### Report generation

This step is to generate two types of reports: numeric report and graphical report. Depending on the data available, the package can generate only graphical report or both reports.

# generate both numeric report

# and      graphical report

> PKshow(myNonmemObj,

table.Colv=TRUE,

table.Rowv=TRUE)

# generate only graphical report

> PKshow()

#### Data cleaning

This step helps to clean R environment and delete figure achieves.

> PKclean()

## Results

One data set from NONMEM was fitted with one-compartment model and utilized for demonstration of PKreport. To illustrate how to use this package, three examples are used. The first example describes how to generate simple graphical report. It works for NONMEM, Monolix, and SAS. The second example demonstrates how to generate a complex report including graphical report and numeric report. It only works for NONMEM 7. The last example focuses on the *nonmem *class and explains how to conveniently retrieve NONMEM 7 output.

### Example 1

This example demonstrates how to generate a simple report (only graphical report and no numerical report). By inputting a simple fitted result, users can generate model diagnostics with graphical report.

> data(pdata)

> var.name <- list(ID="ID", DV="CONC",

   TIME="TIME", PRED="IPRE",

RES="RES", WRES="WRES",

   IDV=c("ISM"), IPRE="PRED",

   ETA=c("ETA1", "ETA2"),

   COV=c("WT","AGE"),

   PARA=c("CL", "V"))

> PKdata(pdata, match.term=var.name)

> PKfigure(pdata, c(3,6,8))

> PKshow()

### Example 2

The NONMEM 7 output directory is in *c:\nnonmem7*, and it includes lst, tab, cov, cor, coi and phi files. We would like to generate a complete report, including both graphical report and numeric report. To create this report, we need to create an instance from the *nonmem *class.

> myclass <- new("nonmem",

output.lst="C:/nonmem7/test.lst",

   output.tab="C:/nonmem7/test.FIT",

   output.dir="C:/nonmem7")

   > var.name <- list(ID="ID", DV="DV",

   TIME="TIME", PRED="PRED",

   RES="RES", WRES=c("WRES"),

   IPRE="IPRED", IDV=c("EVID"),

   ETA=c("ETA5", "ETA1"), COV="TIME",

   PARA=c("ETA2", "ETA3")

   )

> pdata <- myclass@tabdata

> PKdata(data=pdata, match.term=var.name)

> PKfigure(pdata, 3)

> PKfigure(pdata, 7, FALSE)

> PKshow(myclass,

table.Colv=TRUE,

table.Rowv=TRUE)

### Example 3

In this example, we would like to demonstrate how to utilize *nonmem *class to access the NONMEM output.

> non.lst(myclass)

> non.lst.meth(myclass)

[1] "First Order Conditional Estimation

   with Interaction"

> non.lst.objv(myclass)

[1] "2351.625"

# select lines from 50 to 56 in lst file

> exp.data <- non.select(myclass, c(50:56))

> options(scipen = 100)

> PKnum(exp.data)

       [,1]   [,2]       [,3]

[1,]   1  44.80 1000000.00

[2,]   1 410.00 1000000.00

[3,]   0   0.25 1000000.00

[4,]   6  17.00 1000000.00

[5,]   0   0.28 1000000.00

[6,]   0   0.50       0.95

[7,]   0   0.50 1000000.00

> options(scipen = -100)

> PKnum(exp.data)

      [,1]     [,2]    [,3]

[1,] 1e+00 4.48e+01 1.0e+06

[2,] 1e+00 4.10e+02 1.0e+06

[3,] 0e+00 2.50e-01 1.0e+06

[4,] 6e+00 1.70e+01 1.0e+06

[5,] 0e+00 2.80e-01 1.0e+06

[6,] 0e+00 5.00e-01 9.5e-01

[7,] 0e+00 5.00e-01 1.0e+06

## Discussion

In this study, we developed an R package: PKreport as a comprehensive exploratory tool for diagnosing population pharmacokinetic models. It targets audiences working in population pharmacokinetics models, and particularly those professionals who have only basic knowledge of R and lack statistical expertise. PKreport is available in an open-source environment. Based on the questions and suggestions from users, we will continue to update and make it more useful to the community.

As a similar R package to Xpose, PKreport has the following unique features: PKreport is an exploratory report tool rather than a fine-tuned graphical tool. The main objective of this software is to provide a comprehensive view of data, model, and the relationship between them by the automatic pipeline for generating reports. The pharmacologists always hope to use some fancy and specific graphic user interface, which in fact limits and even contradicts the spirit of discovery research. The thought of discovery is the motivation behind this package. Instead of some assumed direction, a systematically full model report helps users to gain deep understanding of the project. On the other hand, Xpose is more like fine-tuned graphical tool to address specific research questions in mind.

In addition, this package automatically generates the R scripts for the plots. This feature allows the experienced users for further amelioration, and thus largely alleviates their repetitive work. For users who do not have expertise in statistics or R, this package can generate all required diagnostic plots with several commands and a few arguments. Anyone has to admit that we can produce any plot and calculate any parameters in R or Matlab, however, the big problem is the time and energy cost. No one wants to repeat it each time for a new model project. In addition, the software design, such as report interface based on web browser, separate stand-alone diagnostic modules, and flexible archive structure for plot management, make it convenient to users.

Furthermore, we proposed and developed a S4 class: *nonmem *to specifically match the new release of NONMEM 7. In the new release, the standard result files are modified and formatted with particular tags to identify various sections. Also, some additional files are generated automatically, including variance-covariance matrix (cov file), correlation matrix (cor file), inverse covariance matrix (coi file) and individual phi parameters and variances (phi file). The new *nonmem *class provides an efficient way to access these output files from NONMEM 7. It includes twelve slots and thirteen methods to access estimation method, analysis information, objective function, title and data for tab, cov, cor, coi, phi files. This package can accept model fit from diverse software, including NONMEM, Monolix, R nlme, etc. By importing the model fit file (for example, tab file in NONMEM) and matching software-specific variables to default modeling variables in metric system, PKreport can explore, visualize and diagnose models from all these software platforms. NONMEM and Monolix both provides some basic diagnostic plots for their fitting results, however, as emphasized before, PKreport servers as a comprehensive exploratory tool and provides a comprehensive way for the data and model. It will be a beneficial and complimentary tool to these software.

## Conclusions

PKreport is an R package that generates a collection of plots and statistics for testing model assumptions, visualizing data and diagnosing models. It provides a flexible and efficient R class to store and retrieve NONMEM output. In addition, it can generate numeric report and graphical report for users to diagnose PopPK models. The general architecture, running environment and statistical methods can be easily extended to include more automatic diagnostics in the development of PopPK models.

## Availability and requirements

• **Project name: **PKreport

• **Project home page**: http://cran.r-project.org/web/packages/PKreport/index.html

• **Operating system(s): **Platform independent

• **Programming language: **R

• **Other requirements: **R packages (lattice, ggplot2)

• **License: **GNU GPL

• **Any restrictions to use by non-academics: **Licence needed

## Competing interests

The authors declare that they have no competing interests.

## Authors' contributions

XS designed the project, developed the package, and drafted the manuscript; JL provided NONMEM 7 data, participated in the development and tested the package. All authors read and approved the final manuscript.

## Pre-publication history

The pre-publication history for this paper can be accessed here:

http://www.biomedcentral.com/1472-6947/11/31/prepub
